# Understanding the molecular basis of EGFR kinase domain/MIG-6 peptide recognition complex using computational analyses

**DOI:** 10.1186/s12859-015-0528-x

**Published:** 2015-03-27

**Authors:** Ninnutt Moonrin, Napat Songtawee, Siriluk Rattanabunyong, Surasuk Chunsrivirot, Wanwimon Mokmak, Sissades Tongsima, Kiattawee Choowongkomon

**Affiliations:** 1 Department of Biochemistry, Faculty of Science, Kasetsart University, 50 Ngam, Wong Wan Rd, Bangkok, Chatuchak 10900 Thailand; 2 Center of Data Mining and Biomedical Informatics, Faculty of Medical Technology, Mahidol University, Bangkok, 10700 Thailand; 3 Department of Biochemistry, Faculty of Science, Chulalongkorn University, Bangkok, Pathum Wan 10330 Thailand; 4 Genetic Engineering Interdisciplinary Program, Graduate School, Kasetsart University, 50 Ngam Wong Wan Rd, Bangkok, Chatuchak 10900 Thailand; 5 Genome Technology Research Unit, National Center for Genetic Engineering and Biotechnology, National Science and Technology Development Agency (NSTDA), 113 Thailand Science Park, Phahonyothin Road, Khlong Nueng, Pathum Thani, Khlong Luang 12120 Thailand; 6 Center for Advanced Studies in Tropical Natural Resources, National Research, University-Kasetsart University, Kasetsart University, Bangkok, Chatuchak 10900 Thailand

**Keywords:** EGFR, Cancer, Inhibitor, Tyrosine kinase, MIG-6 segment1, Molecular dynamics simulations

## Abstract

**Background:**

Epidermal growth factor receptor (EGFR) signalling plays a major role in biological processes, including cell proliferation, differentiation and survival. Since the over-expression of EGFR causes human cancers, EGFR is an attractive drug target. A tumor suppressor endogenous protein, MIG-6, is known to suppress EGFR over-expression by binding to the C-lobe of EGFR kinase. Thus, this C-lobe of the EGFR kinase is a potential new target for EGFR kinase activity inhibition. In this study, molecular dynamics (MD) simulations and binding free energy calculations were used to investigate the protein-peptide interactions between EGFR kinase and a 27-residue peptide derived from MIG-6_s1 segment (residues 336–362).

**Results:**

These 27 residues of MIG-6_s1 were modeled from the published MIG-6 X-ray structure. The binding dynamics were detailed by applying the molecular mechanics Poisson-Boltzmann surface area (MM-PBSA) method to predict the binding free energy. Both van der Waals interactions and non-polar solvation were favorable driving forces for binding process. Six residues of EGFR kinase and eight residues of MIG-6_s1 residues were shown to be responsible for interface binding in which we investigated per residue free energy decomposition and the results from the computational alanine scanning approach. These residues also had higher hydrogen bond occupancies than other residues at the binding interface. The results from the aforementioned calculations reasonably agreed with the previous experimental mutagenesis studies.

**Conclusions:**

Molecular dynamics simulations were used to investigate the interactions of MIG-6_s1 to EGFR kinase domain. Our study provides an insight into such interactions that is useful in guiding the design of novel anticancer therapeutics. The information on our modelled peptide interface with EGFR kinase could be a possible candidate for an EGFR dimerization inhibitor.

## Background

**Figure 1:**
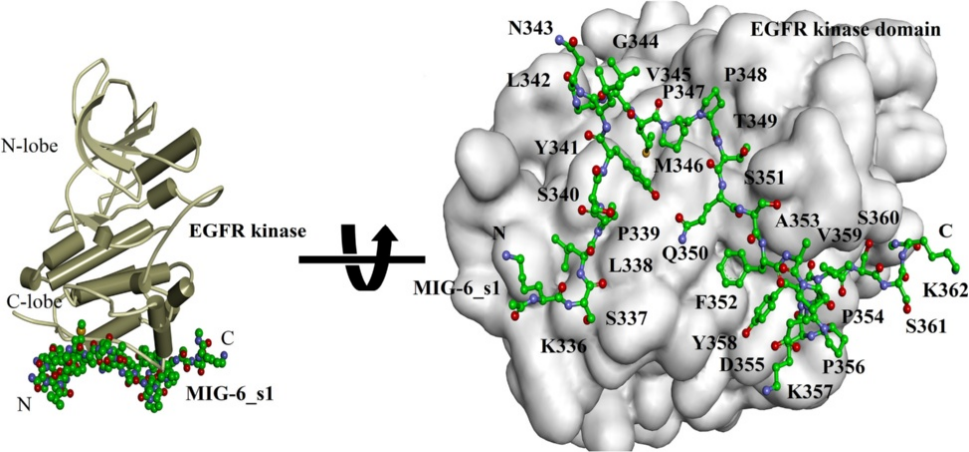
**Cartoon representation of the crystal structure of the EGFR kinase/MIG-6_s1 complex.** The positions of MIG-6_s1 residues were spanned on the EGFR kinase interface.

EGFR, also known as ErbB1 or HER, is a receptor tyrosine kinase that mediates in biological process of normal physiology [1]. An over-expression of EGFR has often been associated with a variety of human cancers that can provide the basic properties required for cancer growth, including cell proliferation, anti-apoptosis, metastasis and angiogenesis [2]. These properties render EGFR an attractive target for cancer therapy. Nonetheless, several reports show that the secondary mutations in EGFR cause anticancer drug resistances [3-5], instigating a requirement of efficient treatments to overcome these resistances. Zhang et al. [6] showed that a new target therapy could be done by the suppression of EGFR activation through an allosteric mechanism. This approach was supported by an association of the endogenous negative-feedback-inhibitor protein, called MIG-6, that regulates the level of EGFR activation [7,8]. The crystal structure of the EGFR kinase/MIG-6 complex showed that a segment of MIG-6 (residues 337–361), called MIG-6_s1 interacts with the C-lobe of EGFR kinase domain (Figure 1) [6]. The binding of MIG-6_s1 to EGFR kinase prevents an asymmetric dimer formation, leading to the inhibition of EGFR activation at micro molar binding affinity level [6]. The detailed analysis of the protein-protein interactions (PPI) at an atomic level of EGFR kinase and MIG-6_s1 is yet limited. Molecular dynamics (MD) simulations and free energy calculation can be used to obtain the knowledge on the dynamics of the structures and the binding energy profiles of the protein complexes in solution [9].

In this study, MD simulations were performed on the EGFR kinase/MIG-6_s1 complex to obtain the knowledge on their binding dynamics and structural changes. The binding free energy calculation by the MM-PBSA method was applied to gain a better understanding on the binding energetics of the complex. The per residue free energy decomposition and alanine scanning methods were used to elucidate the residues important for interface binding and to obtain their energetic contributions. Our results provided a better understanding of the binding interactions between EGFR kinase and MIG-6_s1 peptide. Moreover, such information could be used to improve or develop more efficient anti-cancer drugs.

## Methods

### Model setup and molecular dynamics simulations

The starting structures of EGFR kinase/MIG-6_s1 and the asymmetric kinase dimer complexes were based on the X-ray crystal structure from the protein data bank (PDB). The monomer structures of the EGFR kinase activators (residues 678–959) and MIG-6_s1 (residues 337–362) were used (PDB code: 2RFE) [6], while PDB code: 2GS6 represents the EGFR kinase receiver model. The N- and C-termini of EGFR kinase and MIG-6_s1 were capped with an acetyl group (ACE) and an N-methyl group (NME), respectively [10]. All simulations were performed using AMBER 12 and the ff03 force field [11]. Each system was solvated using the atomistic TIP3P water model [12] in an octahedron truncated box with a buffer distance of 7 Å. Hydrogen atoms were added, and the counter-ions of Na^+^ or Cl^−^ were used to neutralize the system. To remove bad contacts in the crystal structure, we applied three-step minimization to each system before performing the simulations. Positional restraints were applied to the whole system in the first and the second steps with a force constant of 10 kcal/(mol Å^2^) and 2 kcal/(mol Å^2^), respectively. In the third step, all atoms were allowed to move freely without restraints. The energy minimization in each step was carried out using 2,000 cycles of the steepest descent algorithm, followed by 300 cycles of the conjugate gradient algorithm. Each system was heated from 0 to 300K during the 100 ps in an NVT ensemble and was further equilibrated for 500 ps in an NPT ensemble. Then it was simulated for 22 ns in an NPT ensemble at 1 atm and 300K, using the 2 fs integration time step. During the simulations, the particle mesh Ewald (PME) method [13] was used to treat the long-range electrostatics. Moreover, the SHAKE algorithm [14] was applied to eliminate bond-stretching freedom for all bonds involving hydrogen, thereby allowing the use of a 2 fs time step.

### Calculation of binding free energy using the MM-PBSA approach

The MM-PBSA approach [15] was used to calculate protein-protein binding free energies. 300 snapshots were extracted from the last 3 ns of the MD trajectories to perform MM-PBSA. The binding free energy of each snapshot was calculated as follows:

1 $$ \Delta {G}_{\left(\mathrm{bind}\right)}={G}_{\left(\mathrm{complex}\right)}-{G}_{\left(\mathrm{protein}\right)}-{G}_{\left(\mathrm{ligand}\right)} $$

The free energy (*G*) for each molecule can be computed from the following equations:

2 $$ G=\Delta {E}_{\left(\mathrm{gas}\right)}-\Delta {G}_{\left(\mathrm{sol}\right)}-T\varDelta S $$

3 $$ \Delta {E}_{\left(\mathrm{gas}\right)}={E}_{\left(\mathrm{i}\mathrm{n}\mathrm{t}\right)}+{E}_{\left(\mathrm{v}\mathrm{d}\mathrm{w}\right)}+{E}_{\left(\mathrm{e}\mathrm{l}\mathrm{e}\right)} $$

4 $$ {E}_{\left(\mathrm{i}\mathrm{n}\mathrm{t}\right)}={E}_{\left(\mathrm{bond}\right)}+{E}_{\left(\mathrm{angle}\right)}+{E}_{\left(\mathrm{torsion}\right)} $$

5 $$ {G}_{\left(\mathrm{sol}\right),\ \mathrm{PB}}={G}_{\left(\mathrm{PB}\right)}+{G}_{\left(\mathrm{nonpol},\ \mathrm{sol}\right)} $$

6 $$ {G}_{\left(\mathrm{nonpol},\ \mathrm{sol}\right)}=\gamma SASA $$

∆*E*_(gas)_ is the gas-phase energy, which is the sum of the internal (*E*_int_), van der Waals (*E*_vdw_) and Coulomb energies (*E*_ele_) (Eq. 3)_._ Internal energy (*E*_int_) comprises the bond (*E*_bond_), angle (*E*_angle_) and torsion (*E*_torsion_) energies, respectively (Eq.4). The solvation free energy (*G*_sol, PB_) comes from the combination of polar (*G*_PB_) and non-polar contributions (*G*_nonpol, sol_) (Eq.5)*.* Note that *G*_(PB)_ is the polar solvation calculated by the Poisson-Boltzmann (PB) equation with the dielectric constants of 1.0 for solute and 80.0 for solvent. The non-polar component (*G*_nonpol, sol_) is defined in Eq. 6, where the constant γ was set to 0.0072 kcal/mol, while SASA stands for the solvent-accessible-surface area calculated by the linear combination of pairwise overlap [16] model. Finally, −*T*∆*S* in Eq. 2 is an entropy term calculated from the sum of translational, rotational and vibrational components using normal mode analysis. To gain more insights into the residue contributions, the residues of MIG-6_s1 responsible for the binding to the EGFR kinase surface were computed by using the MM-PBSA per residue free energy decomposition method (*mm_pbsa* module) of AMBER12. These calculations were based on the same snapshots as those used in the binding free energy calculations.

### Computational alanine scanning mutagenesis

To investigate the contributions of an individual residue to the binding interface and to identify the “hot-spot” residues that are very important for interface binding, we used computational alanine scanning to identify these key residues of the EGFR kinase/MIG-6_s1 interface as well as the asymmetric dimer interface. Alanine scanning replaces an original residue with alanine; this substitution eliminates the side-chain but does not introduce large conformational changes to alter the mode of binding [17]. The binding free energy of the wild type and mutant were calculated using a post-processing treatment of the *mm_pbsa* module and the difference in their binding free energies was computed using the following equation [10,18]:

7 $$ \Delta \Delta {G}_{\mathrm{binding}}=\Delta {G}_{\mathrm{binding}\kern0.5em \mathrm{of}\kern0.5em \mathrm{wildtype}}-\Delta {G}_{\mathrm{binding}\kern0.5em \mathrm{of}\kern0.5em \mathrm{mutant}} $$

Based on the post-processing energy calculation [19,20], the alanine mutant structure was generated by eliminating the residue side-chain with a hydrogen atom of alanine prior to the calculation. Note that the smallest amino acid, glycine, was not included since it introduces conformational flexibility into the protein backbone. Proline was also not included because its backbone conformation differs from that of alanine [21-23]. The 300 snapshots extracted from the last 3 ns of the MD runs were used in these calculations. If the difference in binding free energy between the wild type and the mutant of a residue (∆∆*G*_binding_) becomes positive, such an alanine substitution is favorable, i.e., this residue is probably not important in the interface binding. However, the negative value of ∆∆*G*_binding_ indicates the unfavorable alanine substitution, i.e., this residue plays significant role in the interface binding [18]. These key residues were clarified as the residues that significantly decrease the binding free energy for at least 2.0 kcal/mol. From literatures, residues with strong binding should reduce the binding free energy greater than or equal to 4 kcal/mol [21,24].

## Results and discussion

### Structural stabilities and flexibilities of the systems

To assess the dynamic stabilities of the systems, we performed MD simulations of EGFR kinase and MIG-6_s1 in both unbound and bound states with explicit water for 22 ns at 300K under 1 atm pressure. The equilibration of MD simulations can be observed from the convergence of the root-mean-square deviation (RMSD) plot, based on the backbone (Cα, N and O) atoms of each snapshot as compared to the initial energy minimized structure.

**Figure 2:**
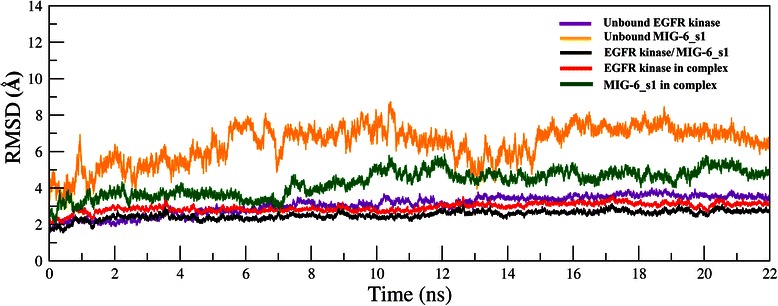
**The Root-Mean-Square Deviation (RMSD) plots of the EGFR kinase /MIG-6_s1 backbones in the bound and unbound states of the 22-ns simulation.** RMSD plots of unbound EGFR kinase and unbound MIG-6_s1 are shown in purple and yellow lines, respectively. For the complex of EGFR kinase/MIG-6_s1, three RMSD plots are presented. The black line represents the RMSD of the entire complex of EGFR kinase/MIG-6_s1. The RMSD plot for only EGFR kinase in the complex is shown in red. The RMSD plot for only MIG-6_s1 in the complex is shown in green.

The RMSD plots of three systems: 1) unbound EGFR kinase 2) unbound MIG-6_s1 and 3) EGFR kinase/MIG-6_s1 complex are shown in Figure 2. For the bound system, beside the plot for overall complex (black line), we also plotted the RMSD values for only EGFR kinase (red line) and for only MIG-6_s1 (green line). The RMSD plots of “unbound EGFR kinase”, “EGFR kinase/MIG-6_s1 complex” and “only EGFR kinase in complex” reached their plateaus after about 2 ns. The RMSD plots of only MIG-6_s1 in the EGFR complex reached its plateau after about 7 ns. These three systems were equilibrated during the 22 ns simulations.

The conformational changes in the bound and unbound simulations were compared (Figure 2). Both bound and unbound EGFR kinase RMSD plots revealed similar patterns with average RMSD value of ~3.0 Å for unbound EGFR and ~2.9 Å for bound. For bound vs. unbound MIG-6_s1, the RMSD plot of bound MIG-6_s1 is more stable than that of unbound state with average RMSD of ~4.2 Å for bound MIG-6_s1 and ~6.4 Å for the unbound MIG-6_s1. It can be seen that the bound MIG-6_s1 was significantly more stable than the unbound state as seen in the lower RMSD fluctuation. Note that the unbound MIG-6_s1 has generally higher RMSD values because MIG-6_s1 is rather short peptide (27 amino acids) and becomes very flexible in solution. This flexible short peptide phenomenon were also presented in [17,25] where the unbound short peptide p53 could not be stabilized in solution but could be bound stably to the binding cleft of MDM2 protein. Thus, from the RMSD results, our MD simulations are reliable enough for further investigation.

The root-mean-square fluctuations (RMSF) of αC atom from the MD simulated structures against the starting structure were calculated to identify the most flexible regions of the protein-peptide structure. The larger RMSF value conveys more flexible region while the lower RMSF value entails the more constrained region.

**Figure 3:**
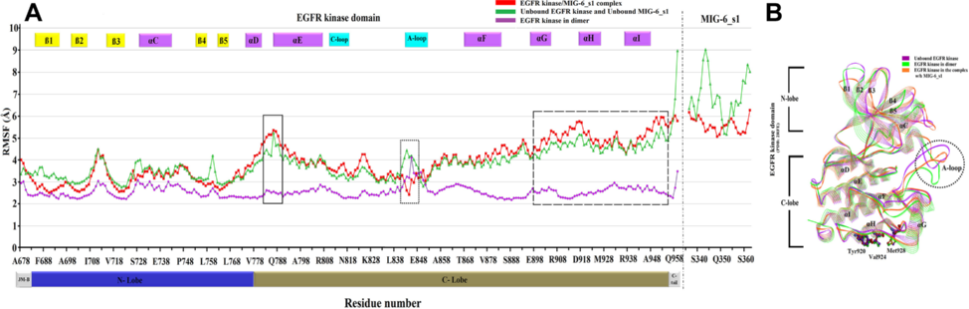
**Graphical representation of root mean square fluctuation (RMSF) and superimposition of EGFR kinase. (A)** The RMSF profiles of EGFR kinase bound MIG-6_s1 (in red), the unbound of EGFR kinase and MIG-6_s1 (in green), and EGFR kinase in the dimeric structure (in purple) with EGFR kinase (PDB: 2GS6). Most flexible regions can be seen as peaks in rectangles. **(B)** the superimposition of EGFR kinase monomers from 1) the unbound EGFR kinase (purple) 2) the EGFR kinase in the binding of MIG-6_s1 complex (orange) and 3) the dimer complex (green). The superimposition revealed the different mobility of the activation loop (A-loop).

The comparison of both unbound EGFR system and EGFR kinase/MIG-6_s1 complex system based on their RMSF profiles (Figure 3A). The RMSF profile of unbound EGFR kinase system (green) showed similar behavior to that of EGFR complex system (red). On the contrary, the RMSF profile of MIG-6_s1 in the unbound state showed much higher fluctuation than that of its complex state. When comparing the RMSF EGFR kinase profile with the dimer complex profile (purple), we observed distinguishable patterns in several regions of the EGFR interface to MIG-6_s1. Within the EGFR N-lobe region, similar RMSF profiles from all three systems were observed; however the corresponding RMSF profiles were lower than the other regions reflecting lower flexibility.

RMSF patterns and their values in the EGFR C-lobe region differ greatly from that of N-lobe region. Particularly, residues from both EGFR kinase/MIG-6_s1 complex and unbound EGFR kinase, especially in the junction αD–αE, and along the three αG, αH and αI helices revealed high RMSF values (reaching 5–6 Ǻ); while the EGFR kinase in dimer complex shows stable RMSF value ~3 Ǻ. We hypothesize that during EGFR dimerization these helices must interact with the juxtamembrane B portion of the EGFR receiver; hence losing the dimerization causes the EGFR activator to have higher RMSF (see unbound EGFR kinase). Furthermore, we observed uncertain fluctuations in the part of Ala840–Gly850, called A-loop, which correlates with the missing residues in the EGFR X-ray crystal structure (Figure 3B) [26].

Toward to the end of the RMSF plots, the magnitude of unbound MIG-6 plot (green) differed greatly from that of complex of EGFR/MIG-6_s1 plot (red). In particular the RMSF of MIG-6_s1 in EGFR complex (red) was about 5–6 Ǻ, while the RMSF of unbound MIG-6_s1 (green) swung much higher. These evidences suggest that the binding of EGFR kinase could help MIG-6_s1 structure to stabilize in complex.

### The conformational changes of the EGFR kinase/MIG-6_s1 complex

**Figure 4:**
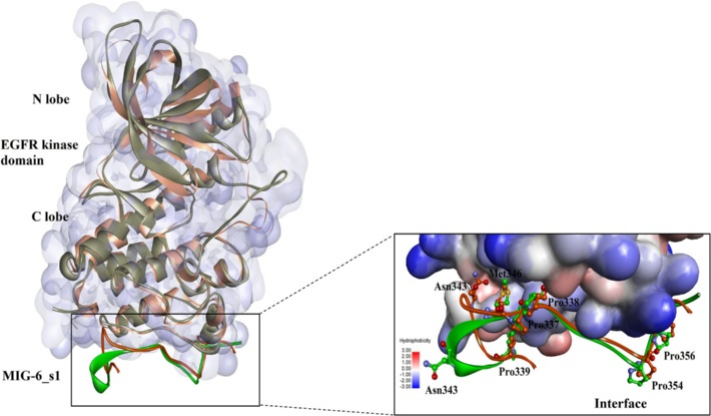
**The superimposition of the average structure from the last 3 ns of simulations (light-pink) and its minimized initial structure (green) of the EGFR kinase /MIG-6_s1 complex.** The positions of Asn343, Met346 and the cluster of prolines of MIG-6_s1 are shown in the ball-and-stick representation.

Twenty-seven residues of MIG-6_s1 (residue 336–362) can bind to the distal surface of the C-lobe in the EGFR kinase domain to prevent the activation of EGFR kinase by asymmetric dimer (Figure 1). To gain a better understanding on the structural differences between the initial structure and the average simulated structure, the superimposition of the minimized initial structure of the EGFR kinase/MIG-6_s1 complex and its average structure from the 300 snapshots of the last 3-ns simulations are shown in Figure 4. The conformations of the average structure and the minimized initial structure were mostly similar, except for the shift in the loop region of MIG-6_s1. Specifically, Asn343 of MIG-6_s1 at the loop region of the average structure shifted its side-chain up closer to the EGFR kinase surface region than that of the minimized initial structure (Figure 4). This contact was not observed previously in the initial structure possibly due to crystal packing contacts in the flexible loop segment [27].

**Figure 5:**
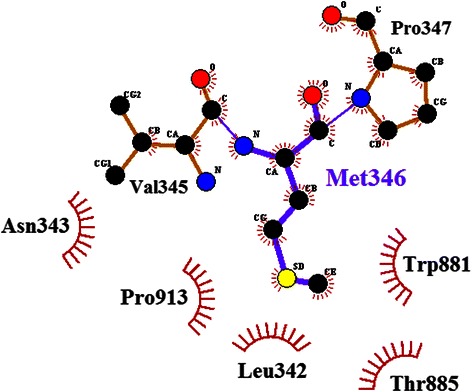
**The interactions of Met346 with its nearby residues in the average structure (plotted by LIGPLOT).** Met346 is shown in the ball-and-stick representation and its neighbours are shown in brown. Carbon, nitrogen and oxygen atom are shown in black, blue and red, respectively. Dark red “eyelashes” represent hydrophobic contacts between Met346 and its neighbours. Black eyelashes correspond to atoms involved in hydrophobic contacts.

**Figure 6:**
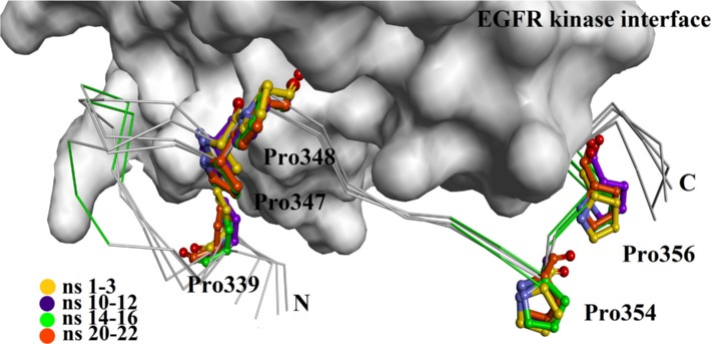
**The superimpositions of the average structures during the 1–3 ns (yellow), 10–12 ns (purple), 14–16 ns (pink), and 20–22 ns (orange) simulations showed the stabilities of prolines over the course of simulations.** The positions of prolines on MIG-6_s1 are indicated on the ball-and-stick representation of EGFR kinase.

In terms of hydrophobic interactions, the hydrophobic pocket of the EGFR kinase interface consists of Leu343, Trp881, Thr885 and Pro913, and the side-chain of Met346 of MIG-6_s1 resided stably in this pocket, as shown in Figure 5 (plotted by LIGPLOT program [28]). Since prolines can affect the rigidity of peptide structures, the conformations of prolines were also analyzed in this study. Five prolines of MIG-6_s1 (Pro347, Pro348, Pro354, Pro356 and Pro339) were in *cis-*configurations and located on the turn. Four of these residues (Pro347, Pro348, Pro354 and Pro356) remained in bent conformation at the interface (Figure 6) [6]. Prolines on the turn might also form interactions between the proline ring and nearby aromatic residues (CH-π) [29] and help stabilize the MIG-6_s1 complex. Similar to prolines in the SH3 domain, the positions of two prolines were relatively fixed to constrain the structural movements [30].

### The calculations of binding free energies

To compute the binding free energies of MIG-6_s1 peptides to EGFR kinase and to gain insights into the peptide-protein binding interactions, the MM-PBSA approach was applied to the 300 snapshots taken from the last 3 ns of the production runs. The calculated results are presented in Table 1. The predicted binding free energy of EGFR kinase/MIG-6_s1 complex was-142.7 kcal/mol. The major favorable components of the MIG-6_s1 binding were the van der Waals term in gas-phase (∆*E*_vdw_ =-120.8 kcal/mol) and the non-polar part of the solvation free energy term (∆*G*_nonpol, sol_ =-84.25 kcal/mol). These two terms resulted in the total favorable non-polar interactions of-205.1 kcal/mol. The highly favorable non-polar part of the free energy might come from the hydrophobic interactions between MIG-6_s1 and EGFR kinase as well as the desolvation of the non-polar groups of MIG-6_s1 from water that aligned them with the binding interface [10]. Such a phenomenon could be seen in several protein-protein interactions including the interaction between MDM2 and p53, where van der Waals interaction was the major contributor to the inhibitor binding originated from the effects of solute-solvent attractive forces on hydrophobic and the dispersion [10,31].

**Table 1:** **The binding free energy of the EGFR kinase/MIG-6_s1 complex and its components calculate using the MM-PBSA method**

**Contribution**	**EGFR kinase/MIG-6_s1**		**EGFR kinase**		**MIG-6_s1**		**Delta**	
	**Mean**	**σ**	**Mean**	**σ**	**Mean**	**σ**	**Mean**	**σ**
*Eele*	-21099.37	5.29	-19392.37	4.9	-1576.62	1.48	-130.38	1.03
*EvdW*	-2536.90	1.24	-2296.92	1.2	-119.22	0.33	-120.75	0.31
*Egas*	-23636.27	5.35	-21689.29	4.96	-1695.84	1.59	-251.13	1.01
*Gsol, np*	2470.43	0.53	2273.28	0.48	281.40	0.21	-84.25	0.16
*Gsol, PB*	-3449.41	4.13	-3449.41	3.87	-351.98	1.08	151.59	0.93
*Gsol*	-978.98	4.03	-975.73	3.8	-70.58	0.94	67.34	0.93
∆*Gcal*							-183.79	0.5
-T∆S	-233583.65	1.09	-3282.65	3.36	-342.04	4.02	41.05	2.08
∆*Gcal* - (-T∆S)							-142.74	-

Mean energies are in kcal/mol, with the corresponding standard errors (σ).

Eele and Evdw are electrostatic and van der Waals contributions in gas phase.

GPB and Gnp are electrostatic and nonpolar contributions in solvation phase.

Δ*Gcal* is total binding free energy.

On the other hand, the favorable columbic term in gas phase (∆*E*_ele_ =-130.4 kcal/mol) was completely cancelled by the unfavorable contribution of the polar part of the solvation free energy (*G*_PB_ = 151.6 kcal/mol), resulting in the total unfavorable electrostatic interactions of 21.2 kcal/mol. This compensation phenomenon was discussed in several studies of protein-protein interactions in solution, including the binding processes of IGF-II/IGF2R and hGH/GHR, giving the polar interactions (∆*E*_ele_ + *G*_PB_) of 31.57 and 134.2 kcal/mol, respectively [10,32]. Moreover, the magnitude of T∆S is in the range of +30 to-40 kcal/mol, being consistent with the previous results [32].

### Free energy decompositions of the interfacial residues

**Figure 7:**
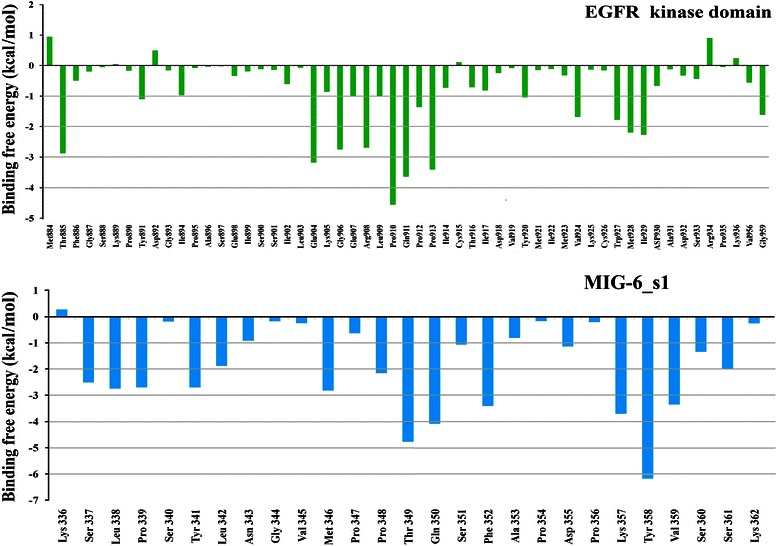
**The per residue free energy decomposition of EGFR kinase and MIG-6_s1**.

**Figure 8:**
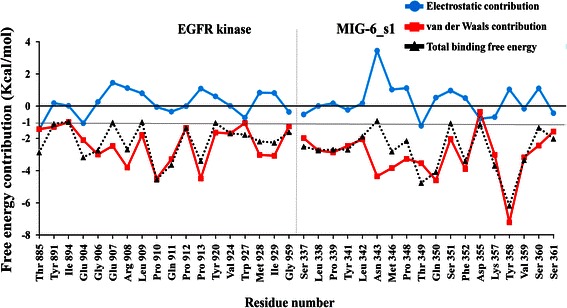
**Per residue free energy decomposition of the residues at the interface of the EGFR kinase and MIG-6_s1 complex.** The van der Waals energy (red line), the sum of the coulombic interactions and the polar part of the solvation free energy (light blue) and the total binding free energy (black dot) for key residues with the values ≤ − 1.0 kcal/mol. All values were given in kcal/mol. Gray line represented the cut off of-1.0 kcal/mol.

The technique of per-residue binding free energy decomposition can reveal the contributions of the key residues responsible for the protein-protein interactions at the interface. The total of 300 snapshots extracted from the last 3 ns of the MD trajectories were decomposed by the MM-PBSA method. The important binding residues of the EGFR kinase/MIG-6_s1 interface were extracted using the residue cut-off at ΔG_tot,PB_ ≤-1.0 kcal/mol (favorable binding) (Figure 7). There were13 important binding residues from MIG-6_s1 and 9 residues from EGFR kinase with binding free energies less than-1 kcal/mol (Figure 8). The 13 residues of MIG-6_s1 comprise Ser337, Leu338, Pro339, Tyr341, Met346, Pro348, Thr349, Gln350, Phe352, Lys357, Tyr358, Val359 and Ser361 whereas the 9 residues of EGFR kinase were Thr885, Glu904, Gly906, Arg908, Pro910, Gln911, Pro913, Met928 and Ile929. Some of these residues, such as Pro910, Thr349, Gln350 and Tyr358, had the values of the binding free energies less than or equal to -4 kcal/mol. Five residues of MIG-6_s1, namely Leu342, Asn343, Ser351, Asp355, and Ser360 and seven residues of EGFR kinase, namely Glu907, Leu909, Pro912, Tyr920, Val924, Trp927 and Gly959, had their binding free energies approximately -1.0 kcal/mol. We further analyzed the binding free energy component of each residue based on the van der Waals energy and the sum of electrostatic contribution in the gas-phase and the polar part of the solvation energy (Figure 8). The results suggested that the main favorable contribution to the binding free energy was essentially the van der Waals interactions. Normally, the favorable electrostatic energies are opposed by the unfavorable polar parts of the solvation free energies (desolvation of the polar groups), resulting in unfavorable contributions. However, some residues had slightly favorable electrostatic contributions (the sum of the electrostatic energies and the polar parts of the solvation free energies) including Thr885, Glu904, Pro910, Gln911, Pro912 and Trp927 of EGFR kinase as well as Ser337, Leu338, Ser340, Tyr341, Val345, Thr349, Pro354, Asp355, Lys357, Val359 and Ser361 of MIG-6_s1.

**Figure 9:**
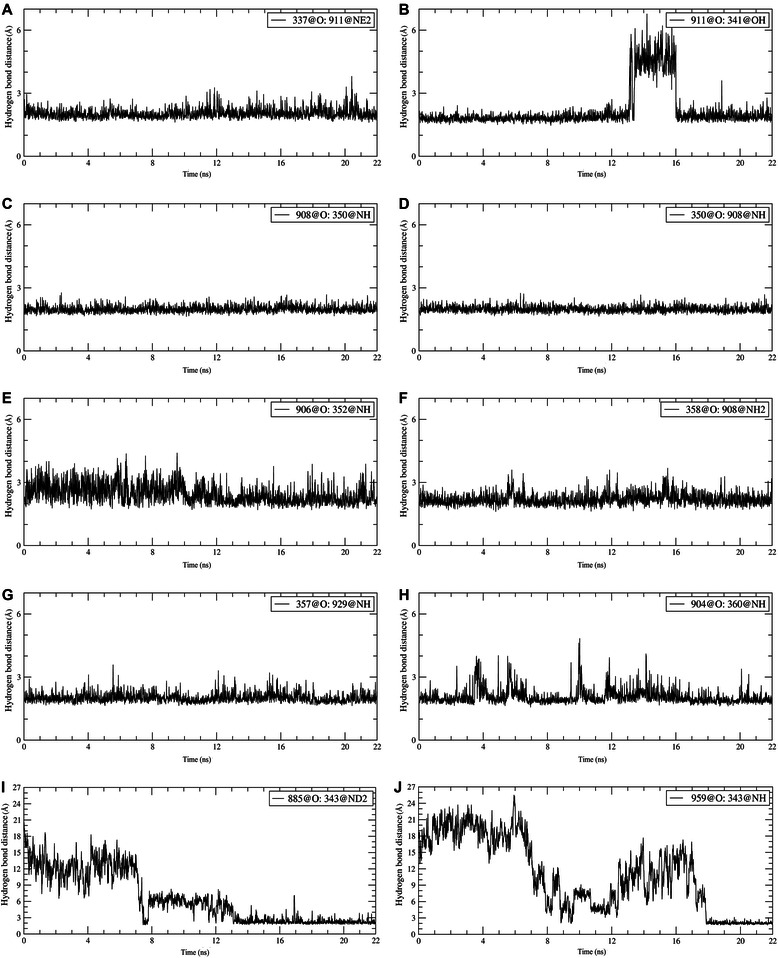
**Hydrogen bond distance profiles. (A)** Between Gln911-NE2-HE21 and Ser337-O. **(B)** Between Tyr341-OH and Gln911-O. **(C)** Between Gln350-NH and Arg908-O. **(D)** Between Arg908-NH and Gln350-O. **(E)** Between Phe352-NH and Gly906-O. **(F)** Between Arg908-NH2-HH22 and Tyr358-O. **(G)** Between Ile929-NH and Lys357-O. **(H)** Between Ser360-NH and Glu904-O. **(I)** Between Asn343-ND2-HD21 and Thr885-O. **(J)** Between Asn343-NH and Gly959-O.

Table 2 shows the information on hydrogen bonds between MIG-6_s1 peptide and EGFR kinase during the last 3 ns of the simulations. These are the hydrogen bonds formed between residues Ser337, Tyr341, Asn350, Phe352, Lys357, Tyr358 and Ser360 of MIG-6_s1 and the residues Glu904, Gly906, Arg908, Gln911 and Ile929 of EGFR kinase. Strong, medium and weak hydrogen-bond interactions were defined as having simulated hydrogen bond occupancy of > 75%, 50–75%, and < 50%, respectively. The hydrogen bond distance profiles of some of the strong hydrogen bond interactions during the 22 ns of the simulations are shown in Figure 9. Most of these hydrogen bonds were stable throughout the entire simulations (Figures 9A, B, C, D, E, F, and G), except for hydrogen bonds shown in Figures 9B, I and J. In particular, the hydrogen bond between Tyr341-OH and Gln911-O was briefly disrupted during 13.5–16 ns of the simulations (Figure 9B). The hydrogen bonds between Asn343-ND2-HD21 and Thr885-O (Figure 9I) and between Asn343-NH and Gly959-O (Figure 9J) were not stably formed until around 13 ns and 18 ns, respectively.

**Table 2:** **Hydrogen bonds found in the last 3-ns of the simulation**

**Donor**	**Acceptor**	**Distance (Å) ± SD**	**Occupancy (%)**
Arg908-NH	Gln350-O	2.916 (0.12)	99.87
Tyr341-OH	Gln911-O	2.838 (0.17)	99.27
Gln350-NH	Arg908-O	2.907 (0.13)	99.20
Ile929-NH	Lys357-O	2.918 (0.15)	98.53
Asn343-NH	Gly959-O	2.994 (0.16)	97.73
Ser360-NH	Glu904-O	2.841 (0.12)	96.83
Gln911-NE2-HE21	Ser337-O	2.954 (0.16)	96.10
Arg908-NH2-HH22	Tyr358-O	2.969 (0.16)	92.73
Asn343-ND2-HD21	Thr885-O	3.063 (0.18)	89.57
Phe352-NH	Gly906-O	3.077 (0.18)	87.43
Gln911-NH	Gln350-OE1	3.034 (0.20)	79.33
Thr349-OG1-HG1	Glu907-OE1	2.697 (0.13)	69.90
Ser361-NH	Glu904-O	3.112 (0.20)	69.37
Ile917-NH	Ser337-O	3.044 (0.17)	51.27
Thr349-OG1-HG1	Glu907-OE2	2.703 (0.18)	24.33

The distance cut-off was 3.5 Å and angle cut-off was 120°.

### Computational alanine scanning technique

Computational alanine scanning is a powerful technique that not only monitors the key residues required for the interactions between the two protein partners at the interface but also computes the energetic contribution to the binding free energy of the individual side-chain based on each alanine mutation [33]. In order to identify the key residues from the 29 residues along the binding interface of the EGFR kinase/MIG-6_s1 complex, we substituted alanine in amino acids having the free energy contribution less than or equal to-1.0 kcal/mol in order to classify them as either “hot spot” or “warm spot” residues. Note that the higher negative value of ∆∆*G* of an alanine point substitution represents the better binding state of the residue. A residue is classified to be very important for binding to the protein partner (hot spot) [21,24,34], if its ∆∆*G* ≤-4 kcal/mol. To a lesser extent, the “warm spot” will be classified if-2 kcal/mol ≥ ∆∆*G* >-4 kcal/mol. In this analysis, proline and glycine residues were not included since their backbone conformations differ from alanine.

**Figure 10:**
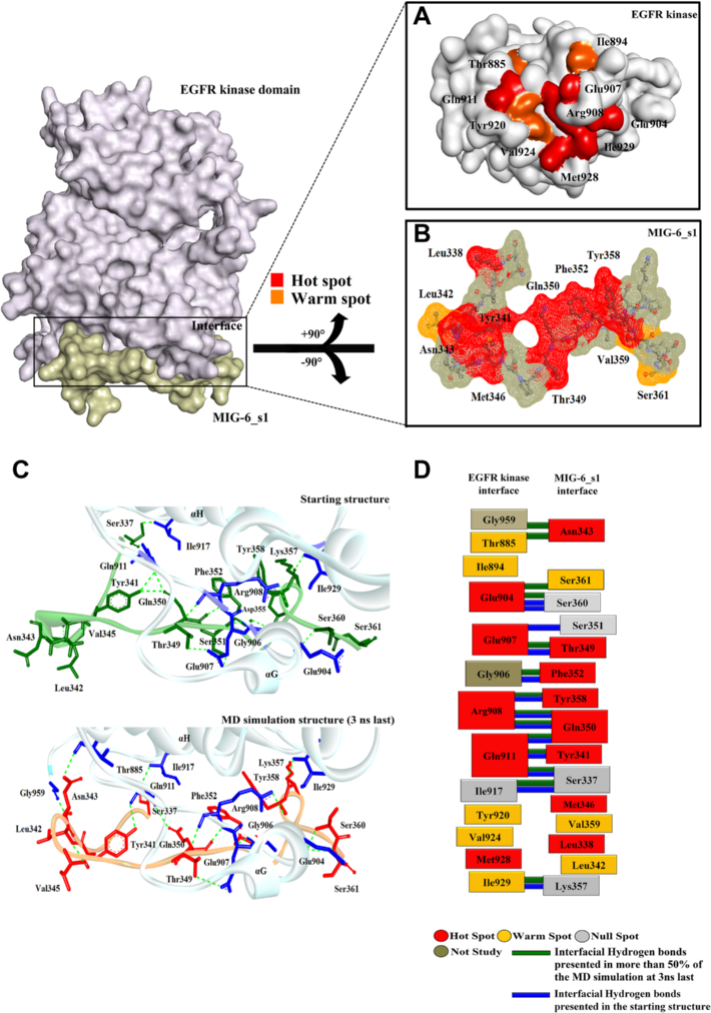
**Schematic representations of the interaction profiles between the EGFR kinase/MIG-6_s1 interfaces. (A)** The key residues of the EGFR kinase surface. **(B)** The key residues of MIG-6_s1. **(C)** Hydrogen bond formation between interfaces presented in the starting structure and the 3 ns last of simulations. Interfacial hydrogen bonds presented in green line with the occupancies higher than 50% **(D)**. The involving of hydrogen bond and key residues between two interfaces presented in the starting structure and the last 3 ns of simulations. The “hot spot residues”, “warm spot”, null spot and not studied residues are shown in red, orange, white and gray respectively. Interfacial hydrogen bonds of the starting structure and MD structure presented in dark green and blue bars, respectively.

The results from the alanine scanning method (Figure 10A and B) and from the per residue free energy decomposition technique (Figure 8) are largely consistent. As determined by the free energy decomposition technique, most of the important residues were also classified as hot spot residues. We showed that there were six “hot spot” residues on EGFR kinase, namely Glu904, Glu907, Arg908, Gln911, Met928 and Ile929; and eight “hot spot” residues on MIG-6_s1, namely Leu338, Try341, Asn343, Met346, Thr349, Gln350, Phe352 and Try358. For the warm spot classification four residues, Thr885, Ile94, Tyr920 and Val924, were on EGFR kinase (Figure 10A) while Leu342, Val359, and Ser361 were on MIG-6_s1 (Figure 10B). However, due to the limitation of alanine scanning, Gly906, Pro910, Pro912, Pro913, Gly959 on EGFR kinase and Pro339, Pro348 on MIG-6_s1 could not be alanine scanned.

Note that by means of per residue free energy decomposition, eight residues (Tyr891, Leu909, Trp927, Ser337, Ser351, Asp355, Lys357 and Ser360) were identified as important residues and not by the alanine scanning approach. Such discrepancies may stem from the differences in the energy cut-offs used in these two methods. Moreover, the alanine scanning method verified only the contributions from the side-chains, while the free energy decomposition analysis considered both the contributions from the side-chains and the backbones. It may be possible that the contributions from the backbones of these residues may be more than those of the side-chains [18]. The common “key” residues identified by both alanine scanning and the free energy decomposition methods, such as Val924, Met346, Phe352 and Try358, are supported by the previous mutagenesis experiment as the alanine replacements of these residues abolished the peptide binding [6].

### The interactions of the key residues at the interface of the EGFR kinase/MIG-6_s1 complex

The common key-residue results from both the per residue binding free energy decomposition and computational alanine scanning technique predicted the most important residues at the binding interface that include six residues of the EGFR kinase and eight residues of the MIG-6_s1peptide segment (Figure 10A and B). These residues have their ∆∆*G* ≤-4 kcal/mol after performing alanine substitution. Both different and consistent interaction profiles between the MD simulations and the starting structures were observed and presented using hydrogen bonding network (Figure 10C). The resulting relationship does not only exhibit their interfacial residues but also some involvements of bonding in the important residues (Figure 10D). The MD simulations mostly reveal stable pattern of hydrogen bonds, with the exception for the bonding of Ser351 to Glu907. Interestingly, these hydrogen bonds were also involved in the important residues on the interface. This suggests that the main contributions to the binding interactions were from the hydrogen bonds.

**Figure 11:**
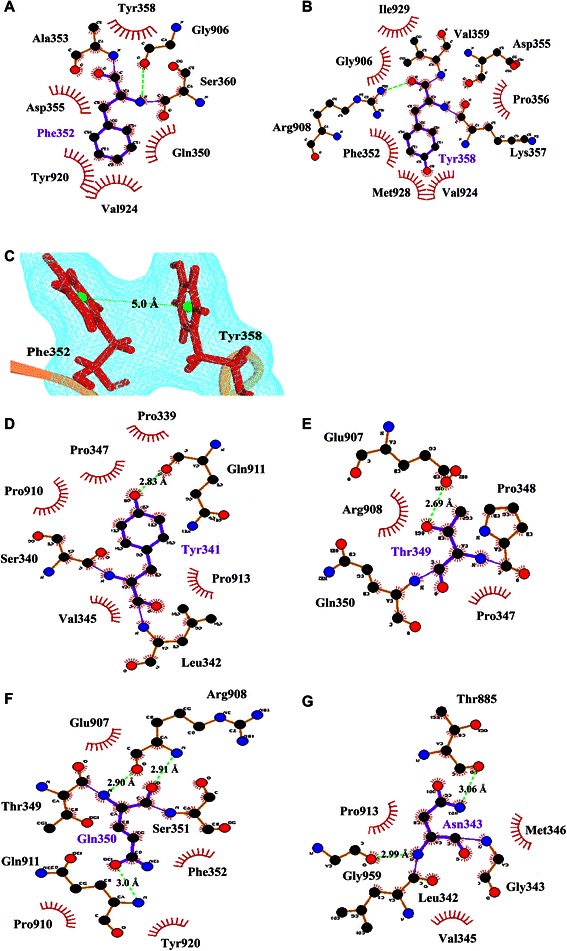
**The interactions of the key residues of MIG-6_s1 with nearby residues. (A)** The hydrophobic interactions and hydrogen bonds of Phe352 and nearby residues. **(B)** The hydrophobic interactions and hydrogen bonds ofTyr358 and nearby residues. **(C)** The π-π interactions between the aromatic rings of Phe352 and Tyr358. **(D)** The hydrogen bond and hydrophobic interactions of Tyr341. **(E)** The hydrogen bonds between polar residues of EGFR kinase Glu907 and Thr349 of MIG-6_s1. **(F)** The hydrogen bonds and hydrophobic interactions of Gln350. **(G)** The hydrogen bonds of Asn343 to Thr885 and Gly959. The important interactions were plotted by LIGPLOT program.

Mutagenesis studies of MIG-6_s1 revealed the importance of Met346, Phe352 and Tyr358 for EGFR kinase binding [6]. With the free energy contribution of approximately-2.8 kcal/mol, Met346 is probably the most important residue of MIG-6_s1 that contributes to the hydrophobic interactions at the interface of the EGFR kinase/MIG-6_s1 complex. The side-chain of Met346 was buried in the hydrophobic pocket consisting of Leu343, Trp881, Thr885 (warm spot) and Pro913 of the EGFR kinase (Figures 4 and 5). The mutating Met346 to Ala346 costs approximately-8.1 kcal/mol of the magnitude of the binding free energy, supporting the importance of its side-chain that engages in the pocket of EGFR kinase. These results were consistent with the crystal structure showing Met346 buried in the hydrophobic pocket of the EGFR kinase, and the experimental results that the mutation of Met346 could abolish EGFR binding [6]. Phe352 and Tyr358 of MIG-6_s1 were also identified in this study as the essential residues for the interface binding with the energetic contributions of about-3.4 and-6.2 kcal/mol, respectively. Their backbones formed hydrogen bonds with hot spot residues, Gly906 and Arg908 (hot spot), respectively, and their side-chains were also buried in the hydrophobic cleft (Figure 11A, B and C). Moreover, the phenyl ring of Phe352 and the phenol ring of Tyr358 formed the paralleled π-π interaction with the distance of 5.0 Ǻ between two centroids (<12.0 Ǻ) (Figure 11C) [29]. Therefore, the mutations of Phe352Ala and Try358Ala could result in the loss of van der Waals contributions from the hydrophobic interactions of their side-chains [6].

Electrostatic and hydrogen bond interactions were also crucial factors for the binding of MIG-6_s1 and EGFR kinase. Tyr341 of MIG-6_s1 was an important residue for the binding of MIG-6_s1 and EGFR kinase with per residue contribution of-1.88 kcal/mol. During the simulations, Tyr341 formed a hydrogen bond with Gln911 (hot spot) and was also buried in the hydrophobic pocket of Pro339, Pro347, Pro910 and Pro913 (Figure 11D). Moreover, the value of ∆∆*G* of the alanine substitution of Tyr341 is about-7.9 kcal/mol, which was influenced by the loss of van der Waals and electrostatic interactions. Another residue important for the binding of this complex is Thr349 of MIG-6_s1. Thr349 formed a hydrogen bond with Glu907 (hot spot) of EGFR kinase (Figure 11E). The value of ∆∆*G* of the alanine substitution of Thr349 is about-7.4 kcal/mol, caused mainly by the loss of electrostatic contributions. Moreover, the backbone of Gln350 formed two hydrogen bonds with the backbone of Arg908 (hot spot), while its side-chain formed not only a hydrogen bond with Gln911 (hot spot) but also formed van der Waals interactions with Pro910 and Try920 (warm spot) (Figure 11F). The value of ∆∆*G* of the alanine substitution of Gln350 is about-4.3 kcal/mol, where van der Waals and electrostatic contributions were lost upon the side-chain mutation.

During the simulations, not only the new hydrogen bond between Ser361 and Glu904 was formed, but the hydrogen bond involving Asn343 at the loop region of MIG-6_s1 was also observed. The side-chain of MIG-6_s1 moved toward the EGFR kinase surface and formed two hydrogen bonds with Thr885 (warm spot) and Gly959 (Figure 11G). These hydrogen bonds started to form around 13 ns and 17 ns and became stable later on with the occupancies of 89.6% and 97.7% for Asn343-Thr885 and Asn343-Gly959, respectively (Figure 9I and J). The ∆∆*G* values of the alanine substitution of Asn343 was-4.9 kcal/mol, where the main contribution came from the loss of van der Waals interactions due to the increased cavity volume after alanine substitution (Table 3). The importance of this residue was also supported by the free energy decomposition per residue analysis of the 9- and 18-residue with the values of -0.86 and -1.11 kcal/mol, respectively. This Asn343 residue could play a very important role for a peptide inhibitor to bind to the EGFR C-lobe interface. However, more studies may be needed to further confirm the importance of this residue.

**Table 3:** **The contribution components of binding free energies upon alanine substitutions**

**Contributions**	**Residue number**									
	Thr885A	Tyr891A	Ile894A	Glu904A	Glu907A	Arg908A	Leu909A	Gln911A	Tyr920A	Val924A
ΔΔ*E*_vdw_	-0.88	-1.22	-1.46	-0.96	-0.67	-4.12	-0.62	-3.12	-2.93	-1.35
ΔΔ*E*_electrostatic_	-0.99	0.38	0.09	-51.15	-60.00	23.34	0.05	-7.21	0.07	0.08
ΔΔ*E*_gas_	-1.85	-0.83	-1.37	-52.11	-60.66	19.22	-0.57	-10.33	-2.86	-1.27
ΔΔ*G*_sol, non pol_	-0.14	-0.24	-1.20	-1.16	-1.73	-0.78	0.00	-1.30	-1.24	-0.93
ΔΔ*G*_sol, PB_	-0.12	-0.18	-0.31	48.52	57.96	-34.08	-0.03	4.15	0.45	-0.55
ΔΔ*G*_solvation_	-0.25	-0.42	-1.51	47.36	56.23	-34.86	-0.03	2.85	-0.79	-1.48
ΔΔ*G*_subtotal_	**-2.10**	**-1.25**	**-2.88**	**-4.75**	**-4.43**	**-15.65**	**-0.60**	**-7.48**	**-3.65**	**-2.75**
	Trp927A	Met928A	Ile929A	Ser337A	Leu338A	Tyr341A	Leu342A	Asn343A	Met346A	Thr349A
ΔΔ*E*_vdw_	-0.70	-3.44	-3.91	-0.43	-3.10	-3.46	-1.40	-4.65	-5.67	-1.10
ΔΔ*E*_electrostatic_	-0.48	-2.63	-1.37	-0.69	0.11	-6.32	-0.05	-3.06	-1.12	-13.24
ΔΔ*E*_gas_	-1.18	-6.07	-5.28	-1.12	-2.99	-9.78	-1.45	-7.71	-7.10	-14.34
ΔΔ*G*_sol, non pol_	1.42	-2.22	-1.54	-0.03	-2.54	-1.20	-1.08	-2.19	-3.12	-1.82
ΔΔ*G*_sol, PB_	0.00	2.99	0.13	1.20	0.73	3.04	0.12	4.98	2.10	8.81
ΔΔ*G*_solvation_	1.42	0.77	-1.14	1.17	-1.81	1.84	-0.96	2.80	-1.02	6.99
ΔΔ*G*_subtotal_	0.24	**-5.30**	**-6.69**	0.05	**-4.80**	**-7.94**	**-2.41**	**-4.91**	**-8.11**	**-7.35**
Contributions	Residue number									
	Gln350A	Ser351A	Phe352A	Asp355A	Lys357A	Tyr358A	Val359A	Ser360A	Ser361A	
ΔΔ*E*_vdw_	-3.82	-0.77	-5.41	-0.18	-2.02	-8.02	-1.56	-1.36	0.15	
ΔΔ*E*_electrostatic_	-6.61	-1.14	0.70	-5.91	1.38	0.14	3.74	-1.52	-8.37	
ΔΔ*E*_gas_	-10.43	-1.91	-4.71	-6.10	-0.64	-7.88	2.18	-2.88	-8.22	
ΔΔ*G*_sol, non pol_	-1.24	-0.07	-3.39	-0.00	-1.44	-4.57	0.41	-0.20	-0.35	
ΔΔ*G*_sol, PB_	7.34	1.03	-0.46	5.13	0.16	2.50	-3.88	2.98	5.37	
ΔΔ*G*_solvation_	6.10	0.96	-3.85	5.13	-1.28	-2.07	-4.29	2.77	5.02	
ΔΔ*G*_subtotal_	**-4.33**	**-0.95**	**-8.56**	**-0.97**	**-1.92**	**-9.95**	**-2.11**	**-0.11**	**-3.02**	

ΔΔ*E*_eJe_ and ΔΔ*E*_vdW_ are electrostatic and van der Waals contributions in gas phase.

ΔΔ*G*_sol,pb_ and ΔΔ*G*_np_ are electrostatic and nonpolar contributions in solvation phase.

Furthermore, the two MIG-6_s1 prolines (Pro339 and Pro348) and those from EGFR kinase domain (Pro910 and Pro913) could also be essential for the interface binding. They not only gave the free energy contributions of about-2.8,-2.2,-4.55 and-3.40 kcal/mol, respectively (these values were larger than those of the other proline residues), but their free energy contributions were more likely to come from the feature of the side-chains that were-2.34,-2.15,-2.71 and-3.19 kcal/mol more than those of their backbones, respectively. Moreover, the proline rings could also form interaction with the side-chain of Tyr341 and Met346. However, the alanine scanning technique could not be done because the mutation of proline to alanine causes structural perturbations of the backbone [21-23].

We also performed alanine scanning on the EGFR kinase binding interface, on ten residues, namely, Thr885, Ile894, Glu904, Glu907, Arg908, Gln911, Tyr920, Val924 Met928 and Ile929. In particular, Thr885 is located within αF of kinase domain and the side-chain pointed to the interface. Thr885 can form hydrogen bonds with Asn343 (warm spot) as well as forming the hydrophobic pocket with side-chain of Met346. However, the loss of ∆∆*G* (-2.1 kcal/mol) was from the loss of van der Waals and electrostatic interactions but they were not large effects.

The negatively charged residues of EGFR kinase interface, Glu904 (αG) could form hydrogen bonds with the uncharged residues of MIG-6_s1, namely Ser360 and Ser361 (warm spot), while the side-chain of Glu907 (at the loop between αG and -αH) formed hydrogen bond with Thr349 side-chain (hot spot). These Glu904 and Glu907 amino acids were classified as hot spot residues due to the mutation of two glutamic acids resulting with the ∆∆*G* costs of-4.75 and-4.43 kcal/mol, respectively. The loss of electrostatic contribution was the major reason of such results.

Likewise, the positively charged Arg908 located between αG and αH possibly made strong hydrogen bonds with the backbone of Gln350 (hot spot) and the side-chain of Tyr358 (hot spot). After alanine mutation, Arg908 was identified as a hot spot residue because of its ∆∆*G-*15.65 kcal/mol that may have been influenced by the increasing cavity volume and the disrupting the conventional hydrogen bonds upon mutating the side-chain. Moreover, the “nitrogen-containing” side chains of Arg908 lining in parallel with the aromatic ring of Tyr358 also formed the cation-π interaction. Although this geometry layout is preferred in protein structures, the mutation of the side-chain could render large favorable electrostatic contribution in the polar solvation [35].

Gln911 (on the loop between αG-αH) was also indicated as a hot spot residue. It disrupted strong hydrogen bonds to Ser337 side-chain upon alanine substitution causing the binding free energy-7.48 kcal/mol, but did not disturb a hydrogen bond of the backbone-side chain between Gln911 and Tyr341. The alignment on αH of Tyr920, Val924 and Met928 showed that they are important to the dimer formation, which were called as “The core of the asymmetric EGFR kinase domain” [36]. These residues not only participate during the hydrophobic interactions that involve residues in the N-lobe of EGFR kinase receiver, but their mutations also abrogated the contribution of the asymmetric dimer [6,36].

These experimental results align with our *in silico* predictions, these residues were superimposed to those in different structures as shown in the RMSF plots (Figure 3) demonstrating the stability of their backbones. Moreover, we took the EGFR kinase activator interface when binding to MIG-6_s1 and compared with the one when binding to the EGFR kinase receiver. The important of these residues were observed on the above EGFR kinase activator interfaces. After alanine mutations, the loss of van der Waals interaction could originate from the disruption of hydrophobic interaction to their partner proteins. The binding energies of Tyr920, Val924 and Met928 values are-3.65,-2.75, and-5.30 kcal/mol (when binding to MIG-6_s1) and-3.46,-4.98, and-6.07 kcal/mol (when binding to receiver kinase), respectively. The sharing residues between two interfaces of the activator entail that MIG-6_s1 might bind the EGFR kinase activator in the manner similar to a binding of the receiver kinase, even though the function of MIG-6_s1 was opposed to the receiver kinase [6,37].

## Conclusions

In this study, the 22 ns MD simulations of EGFR kinase/MIG-6_s1 complex was performed in order to provide more understanding about the binding of MIG-6_s1 peptide to the C-lobe of EGFR kinase that is an important target in cancer therapy. Using both per residue free energy decomposition and computational alanine scanning techniques, we found that the Asn343 residue situates on a flexible loop of MIG-6_s1. This particular loop was absent in the original X-ray crystal structure. Furthermore, the underlying loop potentially renders Asn343 a key residue to bind to EGFR kinase. This finding requires further experimental validation to better our understanding about Asn343 role in the protein-protein interaction.

In addition, we used the above energy calculation techniques to identify the hot spot residues (six residues on EGFR kinase and eight residues on MIG-6_s1) that play significant role in the binding between the two proteins. Particularly, the alanine scanning technique showed that the alanine mutations on these hot spot residues would worsen the EGFR kinase/MIG-6_s1 interaction. This technique also revealed the other important residues but to a lesser extent, called warm spot residues that might be worth to further investigate their binding roles. These key amino acids proposed in this work should play vital role for a peptide-based inhibitor to prevent the EGFR asymmetric dimer formation. This *in silico* study, hence, provides valuable information for designing new peptide-based cancer drugs.

## Abbreviations

EGFR: Epidermal growth factor receptor
MIG-6_s1: Mitogen induce gene 6 segment1
MM-PBSA: Molecular mechanics/poisson-boltzmann surface area
RMSD: Root-mean-square-deviations

## References

[1] Hynes NE, MacDonald G (2009). ErbB receptors and signaling pathways in cancer. Curr Opin Cell Biol.

[2] Sebastian S, Settleman J, Reshkin SJ, Azzariti A, Bellizzi A, Paradiso A (2006). The complexity of targeting EGFR signalling in cancer: from expression to turnover. Biochim Biophys Acta.

[3] Wang DD, Zhou W, Yan H, Wong M, Lee V (2013). Personalized prediction of EGFR mutation-induced drug resistance in lung cancer. Sci Rep.

[4] Friedman R (2013). Drug resistance missense mutations in cancer are subject to evolutionary constraints. PLoS One.

[5] Yun CH, Mengwasser KE, Toms AV, Woo MS, Greulich H, Wong KK (2008). The T790M mutation in EGFR kinase causes drug resistance by increasing the affinity for ATP. Proc Natl Acad Sci U S A.

[6] Zhang X, Pickin KA, Bose R, Jura N, Cole PA, Kuriyan J (2007). Inhibition of the EGF receptor by binding of MIG6 to an activating kinase domain interface. Nature.

[7] Zhang YW, Vande Woude GF (2007). Mig-6, signal transduction, stress response and cancer. Cell Cycle.

[8] Zhang YW, Staal B, Su Y, Swiatek P, Zhao P, Cao B (2007). Evidence that MIG-6 is a tumor-suppressor gene. Oncogene.

[9] Yang Y, Liu H, Yao X (2012). Understanding the molecular basis of MK2-p38alpha signaling complex assembly: insights into protein-protein interaction by molecular dynamics and free energy studies. Mol BioSyst.

[10] Guo J, Wang X, Sun H, Liu H, Yao X (2012). The molecular basis of IGF-II/IGF2R recognition: a combined molecular dynamics simulation, free-energy calculation and computational alanine scanning study. J Mol Model.

[11] Case DA, Cheatham TE, Darden T, Gohlke H, Luo R, Merz KM (2005). The Amber biomolecular simulation programs. J Comput Chem.

[12] Jorgensen WL, Chandrasekhar J, Madura JD, Impey RW, Klein ML (1983). Comparison of simple potential functions for simulating liquid water. J Chem Physics.

[13] Darden T, York D, Pedersen L (1993). Particle mesh Ewald: an n⋅log (N) method for Ewald sums in large systems. J Chem Physics.

[14] Miyamoto S, Kollman PA (1992). Settle: an analytical version of the SHAKE and RATTLE algorithm for rigid water models. J Comput Chem.

[15] Kollman PA, Massova I, Reyes C, Kuhn B, Huo S, Chong L (2000). Calculating structures and free energies of complex molecules: combining molecular mechanics and continuum models. Acc Chem Res.

[16] Weiser J, Shenkin PS, Still WC (1999). Approximate atomic surfaces from linear combinations of pairwise overlaps (LCPO). J Comput Chem.

[17] Zhong H, Carlson HA (2005). Computational studies and peptidomimetic design for the human p53-MDM2 complex. Proteins.

[18] Li T, Froeyen M, Herdewijn P (2008). Computational alanine scanning and free energy decomposition for E. coli type I signal peptidase with lipopeptide inhibitor complex. J Mol Graph Model.

[19] Bradshaw RT, Patel BH, Tate EW, Leatherbarrow RJ, Gould IR (2011). Comparing experimental and computational alanine scanning techniques for probing a prototypical protein-protein interaction. Protein Eng Des Sel.

[20] Saiz-Urra L, Cabrera MA, Froeyen M (2011). Exploring the conformational changes of the ATP binding site of gyrase B from Escherichia coli complexed with different established inhibitors by using molecular dynamics simulation: protein-ligand interactions in the light of the alanine scanning and free energy decomposition methods. J Mol Graph Model.

[21] Morrison KL, Weiss GA (2001). Combinatorial alanine-scanning. Curr Opin Chem Biol.

[22] Bullock BN, Jochim AL, Arora PS (2011). Assessing helical protein interfaces for inhibitor design. J Am Chem Soc.

[23] Massova I, Kollman PA (1999). Computational alanine scanning to probe protein − protein interactions: a novel approach to evaluate binding free energies. J Am Chem Soc.

[24] Sousa SF, Tamames B, Fernandes PA, Ramos MJ (2011). Detailed atomistic analysis of the HIV-1 protease interface. J Phys Chem B.

[25] Espinoza-Fonseca LM, Trujillo-Ferrara JG (2006). Conformational changes of the p53-binding cleft of MDM2 revealed by molecular dynamics simulations. Biopolymers.

[26] Red Brewer M, Choi SH, Alvarado D, Moravcevic K, Pozzi A, Lemmon MA (2009). The juxtamembrane region of the EGF receptor functions as an activation domain. Mol Cell.

[27] Bonvin AM, Brunger AT (1995). Conformational variability of solution nuclear magnetic resonance structures. J Mol Biol.

[28] Wallace AC, Laskowski RA, Thornton JM (1995). LIGPLOT: a program to generate schematic diagrams of protein-ligand interactions. Protein Eng.

[29] McGaughey GB, Gagne M, Rappe AK (1998). pi-Stacking interactions: alive and well in proteins. J Biol Chem.

[30] Stein A, Aloy P (2008). Contextual specificity in peptide-mediated protein interactions. PLoS One.

[31] Niu RJ, Zheng QC, Zhang JL, Zhang HX (2013). Molecular dynamics simulations studies and free energy analysis on inhibitors of MDM2-p53 interaction. J Mol Graph Model.

[32] Huo S, Massova I, Kollman PA (2002). Computational alanine scanning of the 1:1 human growth hormone-receptor complex. J Comput Chem.

[33] Moreira IS, Fernandes PA, Ramos MJ (2007). Hot spots–a review of the protein-protein interface determinant amino-acid residues. Proteins.

[34] Pons J, Rajpal A, Kirsch JF (1999). Energetic analysis of an antigen/antibody interface: alanine scanning mutagenesis and double mutant cycles on the HyHEL-10/lysozyme interaction. Protein Sci.

[35] Crowley PB, Golovin A (2005). Cation-pi interactions in protein-protein interfaces. Proteins.

[36] Zhang X, Gureasko J, Shen K, Cole PA, Kuriyan J (2006). An allosteric mechanism for activation of the kinase domain of epidermal growth factor receptor. Cell.

[37] Jura N, Endres NF, Engel K, Deindl S, Das R, Lamers MH (2009). Mechanism for activation of the EGF receptor catalytic domain by the juxtamembrane segment. Cell.

